# Two phase kinetics of the inflammatory response from hepatocyte-peripheral blood mononuclear cell interactions

**DOI:** 10.1038/s41598-019-44840-w

**Published:** 2019-06-10

**Authors:** Audrey Beringer, Jennifer Molle, Birke Bartosch, Pierre Miossec

**Affiliations:** 1 Immunogenomics and Inflammation research Unit EA 4130, University of Lyon, Lyon, France; 2 Cancer research center Lyon, INSERM U1052 and CNRS 5286, University of Lyon, Lyon, France

**Keywords:** Cytokines, Translational research

## Abstract

Active liver diseases are characterized by an infiltration of inflammatory immune cells, which interact locally with hepatocytes. Co-cultures between non- and -activated human peripheral blood mononuclear cells (PBMCs) and human hepatoma HepaRG cells were used to determine the role of these cell interactions in the inflammatory response. At the early stage, PBMC-HepaRG cell interactions increased mRNA expression and/or secretion of IL-6, IL-8, CCL-20 and MCP-1, in part through direct cell contact and the induction was higher in PHA-activated conditions. The pro-inflammatory cytokines IL-17 and/or TNFα contributed to the increase of IL-6 and IL-8 secretion. HepaRG cells modulated T cell polarization by increasing Th1 cell transcription factor expression and by reducing CD3^+^ CD4^+^ IL-17^+^ cell frequency when PBMCs were activated with PHA. At a later stage, the presence of HepaRG cells inhibited PHA-induced HLA-DR expression on PBMCs, and PBMC proliferation. In contrast, the presence of skin fibroblasts had no effect of PBMC proliferation induced by PHA. After a first pro-inflammatory phase, PBMC-HepaRG cell interactions may down-regulate the immune response. The PBMC-hepatocyte interactions can thus participate first to the initiation of hepatitis and later to the maintenance of immune tolerance in liver, possibly contributing to chronicity.

## Introduction

Liver is perceived as a metabolic organ, but it is also a central intersection point of the immune system. Receiving 80% of its blood supply from the gut, liver is constantly exposed to environmental toxins, dietary and bacterial products via the portal vein^1^. It plays also an important role in the initiation of the acute-phase response by producing acute-phase proteins. The structural organization of the liver, the hepatic cell repertoire and its “buffer” function between the gut content and systemic inflammation create an unique environment, which determines the balance between inflammation and immunosuppression^1^.

Because liver transplantation is rather better tolerated and requires less immunosuppressive therapy compared to other allogeneic grafts such as skin graft, liver is often seen as an immunologically tolerant organ^2,3^. In addition, it mediates systemic tolerance since liver graft protects other transplanted organs from rejection^4–6^.

During any liver injury and inflammation, immune cells migrate to the liver and interact with liver resident cells through soluble and contact-dependent interactions, specifically with hepatocytes, the most abundant cell population in liver. In response, hepatocytes secrete mediators and cytokines, that in turn modulate the immune cell response^7^. In previous studies, it was observed that interactions between cells constituting the tissues and the infiltrated immune cells increase the inflammatory response in different cell culture models including synovium or skin fibroblasts^8^. However, the role of these cell interplays is still poorly understood in liver, where the immune regulation is known to be different.

The objective of this study was to assess the role of the interactions between immune cells and hepatocytes on the inflammatory response by using co-cultures with human cells. PBMC-hepatocyte interactions enhanced the release of pro-inflammatory cytokines and chemokines. In contrast, hepatocytes reduced PBMC HLA-DR expression and proliferation in activated conditions. PBMC-hepatocyte interactions can thus participate first to the initiation of hepatitis and later, also to the maintenance of immune tolerance in liver.

## Results

### PBMC-hepatocyte interactions induce IL-6 production in activated conditions

**Figure 1:**
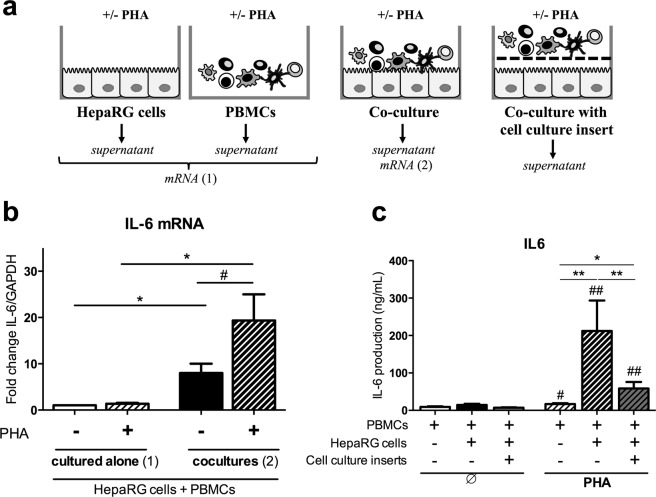
PBMC-HepaRG cell interactions increase expression and/or secretion of IL-6 in activated condition mainly through direct cell-cell contact. (**a**) Human PBMCs and HepaRG cells were cultured alone or in co-cultures with or without cell culture inserts at a ratio of 5 PBMCs: 1 HepaRG cell in presence or not of PHA. (**b**) IL-6 mRNA expression at 24 h in PBMCs and HepaRG cells were expressed as fold changes compared to non-activated conditions with PBMCs and HepaRG cells cultured alone. (**c**) IL-6 supernatant levels were quantified by ELISA at 48 h in PBMC monocultures, PBMC-HepaRG cell co-cultures with or without cell culture inserts. Data are the mean of 7 to 9 independent experiments ± SEM; Mann Whitney test, *p < 0.05, **p < 0.01 versus co-culture conditions; ^#^p < 0.05 and ^##^p < 0.01 versus non-activated conditions.

Acute-phase proteins such as CRP are mainly produced by hepatocytes in response to pro-inflammatory cytokines, specifically IL-6^9^. In liver, hepatocytes interact and establish cell-cell contacts with PBMCs^10^. The effects of these interactions on IL-6 mRNA expression and secretion were studied using a co-culture system with PBMCs from healthy donors and the human HepaRG cell line (Fig. 1a). Because it is not possible to separate PBMCs from HepaRG cells after they have been co-cultured, total mRNA from both cells was recovered. For monocultures, mRNA from HepaRG cell cultures and PBMC cultures were therefore pooled. This way, the role of cell interactions on mRNA expression can be assessed. When PBMCs and HepaRG cells were cultured together, IL-6 mRNA levels were higher (>8.0-fold, p < 0.05) than when cells were cultured separately, and such increase was even stronger in PHA activated conditions (>19-fold, p < 0.05) (Fig. 1b). Regarding IL-6 secretion, supernatant levels were similar between PBMC monocultures and co-cultures in non-activated conditions. In contrast, PHA activation strongly enhanced IL-6 release in PBMC-HepaRG cell co-cultures compared to the other conditions (p < 0.05). IL-6 level was 18-fold higher in co-cultures versus PBMCs alone in PHA-stimulated cultures. In addition, the use of cell culture inserts to avoid direct cell-cell contacts but not the circulation of soluble factors reduced by 72% (p < 0.01) IL-6 secretion compared to co-cultures without inserts (Fig. 1c). PBMC-HepaRG cell interactions thus induced a strong IL-6 secretion in PHA-activated conditions and direct cell-cell contact had an important contribution.

### PBMC-hepatocyte interactions increase IL-8, CCL20 and MCP-1 chemokine expression/secretion

**Figure 2:**
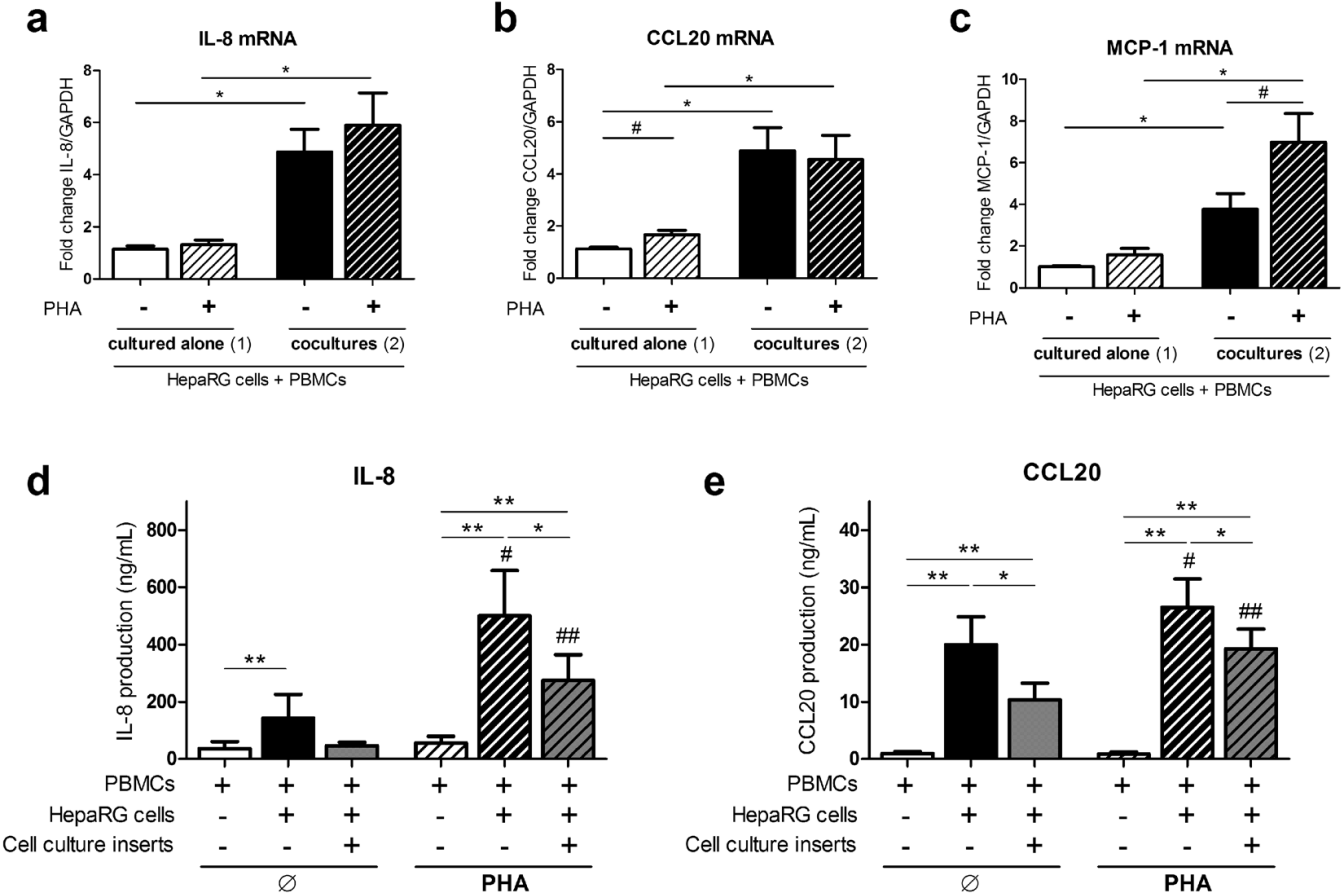
PBMC-HepaRG cell interactions increase expression and/or secretion of IL-8, CCL20 and MCP-1 chemokines. Human PBMCs and HepaRG cells were cultured alone or in co-cultures with or without cell culture inserts at a ratio of 5 PBMCs: 1 HepaRG cell in presence or not of PHA. (**a**–**c**) IL-8 and CCL20 mRNA expression at 24 h and MCP-1 mRNA expression at 8 h in PBMCs and HepaRG cells were expressed as fold changes compared to non-stimulated conditions with PBMCs and HepaRG cells cultured alone. (**d**,**e**) IL-8 and CCL20 supernatant levels were quantified by ELISA after 48 h of culture in PBMC monocultures, PBMC-HepaRG cell co-cultures with or without cell culture inserts. Data are the mean of 7 to 8 independent experiments ± SEM; Mann Whitney test, *p < 0.05, **p < 0.01 versus co-culture conditions; ^#^p < 0.05 and ^##^p < 0.01 versus non-stimulated conditions.

Chemokine release induces immune cell recruitment that is crucial for the inflammatory response. Different inflammatory infiltrates are found in biopsies of acute or chronic liver diseases^11^. IL-8 is involved in neutrophil recruitment, as in acute-liver injury whereas chemokine (C-C motif) ligand 20 (CCL20) and monocyte chemoattractant protein (MCP-1) attract dendritic cells, T cells and monocytes, as in chronic liver injury. The effect of PBMC-HepaRG cell interactions on IL-8, CCL20 and MCP-1 chemokines was thus investigated. mRNA expression of IL-8, CCL20 and MCP-1 was up-regulated (4.9-; 4.6- and 3.8-fold, respectively, p < 0.05) in PBMC-HepaRG cell co-cultures compared to PBMCs and HepaRG cells cultured alone (Fig. 2a–c). PHA activation increased MCP-1 mRNA levels by 1.8-fold (p < 0.05) in co-cultures (Fig. 2c). In contrast, IL-8 and CCL20 mRNA levels in co-cultures were not significantly different between non-activated and PHA-activated co-cultures (Fig. 2a,b). Quantification of IL-8 and CCL20 in supernatants confirmed the increased induction of IL-8 and CCL20 by PBMC-HepaRG cell interactions compared to PBMCs alone (p < 0.01) and HepaRG cells alone (17.5 ng/mL for IL-8 and 3.6 ng/mL for CCL20, p < 0.01, data not shown) in both non- and PHA-activated conditions (Fig. 2d,e). PHA activation enhanced IL-8 and CCL-20 secretion in co-cultures (p < 0.05). Inhibition of the direct cell contacts with inserts reduced IL-8 and CCL20 release by 45% and 19%, respectively (p < 0.05) in activated conditions (Fig. 2d,e). The contribution of direct cell-cell contacts to IL-8 and CCL20 release was lower than that of IL-6 in the same conditions (72%) (Fig. 1b). Therefore, both cell contacts and soluble factors between PBMCs and HepaRG cells had a major contribution to the release of IL-8 and CCL20.

### IL-17 and TNFα contribute to the induction of IL-6 and IL-8 secretion in co-cultures

**Figure 3:**
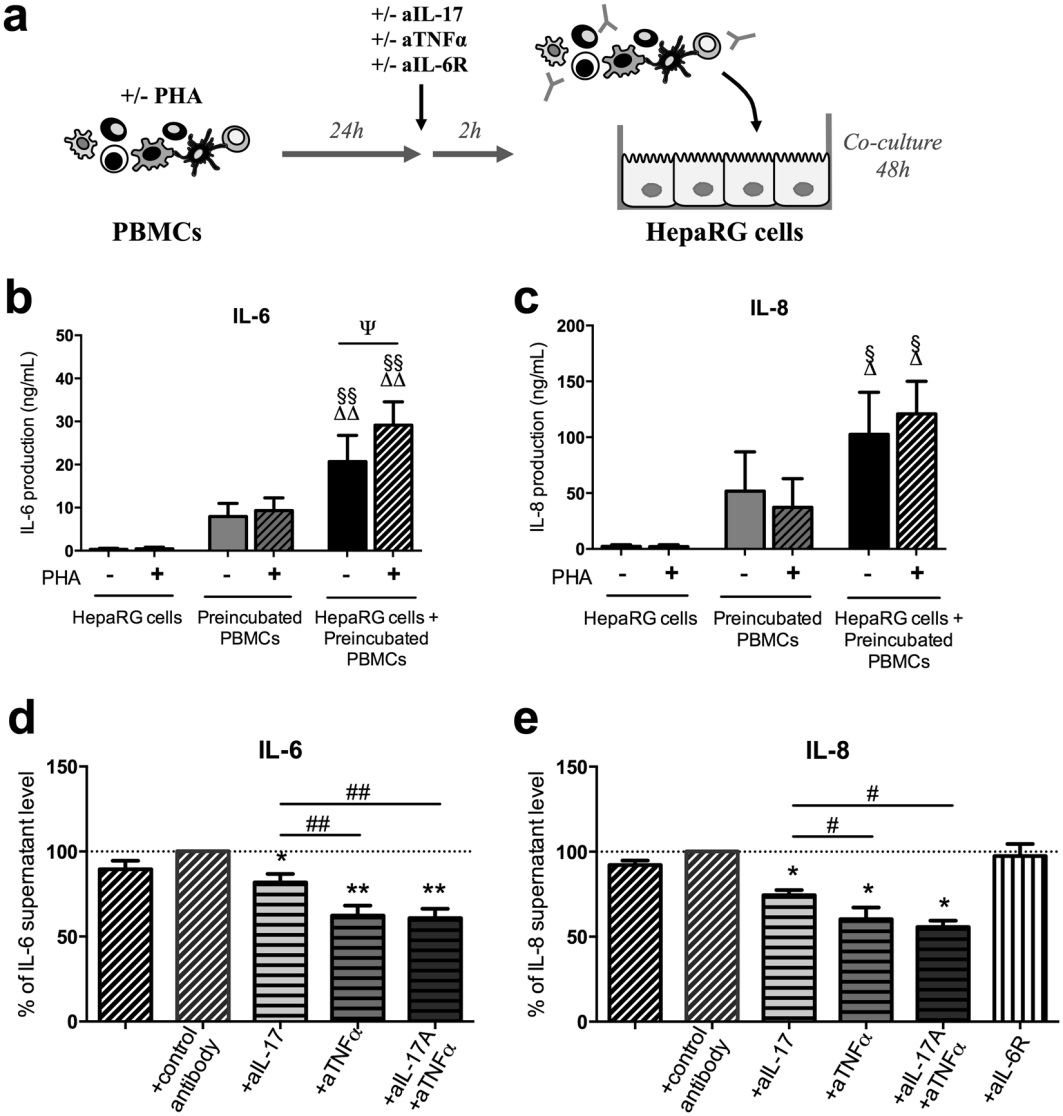
IL-17 and TNFα contribute to the induction of IL-6 and IL-8 secretion by the PBMC-HepaRG cell interactions. (**a**) Human PBMCs were incubated for 24 h in presence or not of PHA and then exposed or not to anti-IL-17 (aIL-17a) and/or anti-TNFα (aTNFα) or anti-IL-6 receptor (aIL-6R) or control antibody during 2 h before being added to HepaRG cells. IL-6 and IL-8 supernatant levels were quantified by ELISA after 48 h of co-cultures. (**b**,**c**) Co-cultures between HepaRG cells and pre-incubated PBMCs increased IL-6 and IL-8 secretion compared to HepaRG cells alone or pre-incubated PBMCs alone. (**d**,**e**) Data are expressed as IL-6 or IL-8 supernatant level percentages compared to the PHA-activated PBMC–HepaRG cell co-cultures in presence of the control antibody. Data are the mean of 7 to 8 independent experiments ± SEM; Mann Whitney test, Δp < 0.05 and ΔΔp < 0.01 versus HepaRG cell alone; ^§^p < 0.05 and ^§§^p < 0.01 versus preincubated PBMC alone; ψp < 0.05 versus PHA condition; *p < 0.05 and **p < 0.01 versus the PHA-stimulated co-culture conditions with the control antibody; ^#^p < 0.05 and ^##^p < 0.01 versus the PHA-stimulated co-culture conditions with the anti-IL-17.

IL-17 and tumor necrosis factor-α (TNFα) are two pro-inflammatory cytokines known to induce, in synergy, a massive IL-6 and IL-8 production by hepatocytes^12^. As shown above in Figs 1c and 2d, PBMC-HepaRG cell interactions enhanced the secretion of IL-6 and IL-8 and part of this effect was mediated through soluble factor exchanges. To determine the contribution of IL-17 and TNFα produced by PBMCs on IL-6 and IL-8 release in co-cultures, PBMCs first activated with PHA for 24 h were exposed to blocking antibodies for IL-17 and/or TNFα and then added to HepaRG cell cultures (Fig. 3a). This PBMC pre-incubation step was used to better mimic the *in vivo* conditions in chronic inflammatory disorders in which PBMCs are pre-activated before reaching hepatocytes and other resident tissue cells. As expected, interactions between pre-incubated PBMCs and HepaRG cells increased IL-6 and IL-8 secretion compared to PBMCs alone or HepaRG cells alone (Fig. 3b,c). Neutralization of IL-17, TNFα or both reduced significantly the production of IL-6 by 18%, 38% and 39% and IL-8 by 26%, 39% and 44%, respectively versus the condition with the control antibody. IL-6 and IL-8 secretion was lower in presence of anti-TNFα alone or the combination of anti-IL-17 and anti-TNFα compared to anti-IL-17 alone (p < 0.01 for IL-6 and p < 0.05 for IL-8) (Fig. 3d,e). Therefore, the use of both anti-IL-17 and anti-TNFα antibodies had no additive or synergistic inhibitory effects on IL-6 and IL-8 release. Consistent with our prior experiments with HepaRG cell monocultures^12^, blockade of IL-6 had no effect on IL-8 release in co-cultures (Fig. 3e). IL-17 and TNFα contributed thus to the induction of IL-6 and IL-8 secretion in PBMC-HepaRG cell co-cultures but the combination of IL-17 and TNFα inhibitors had no additive effect on IL-6 and IL-8 inhibition.

### PBMC-HepaRG cell interactions modulate T cell polarization in PHA-activated conditions

**Figure 4:**
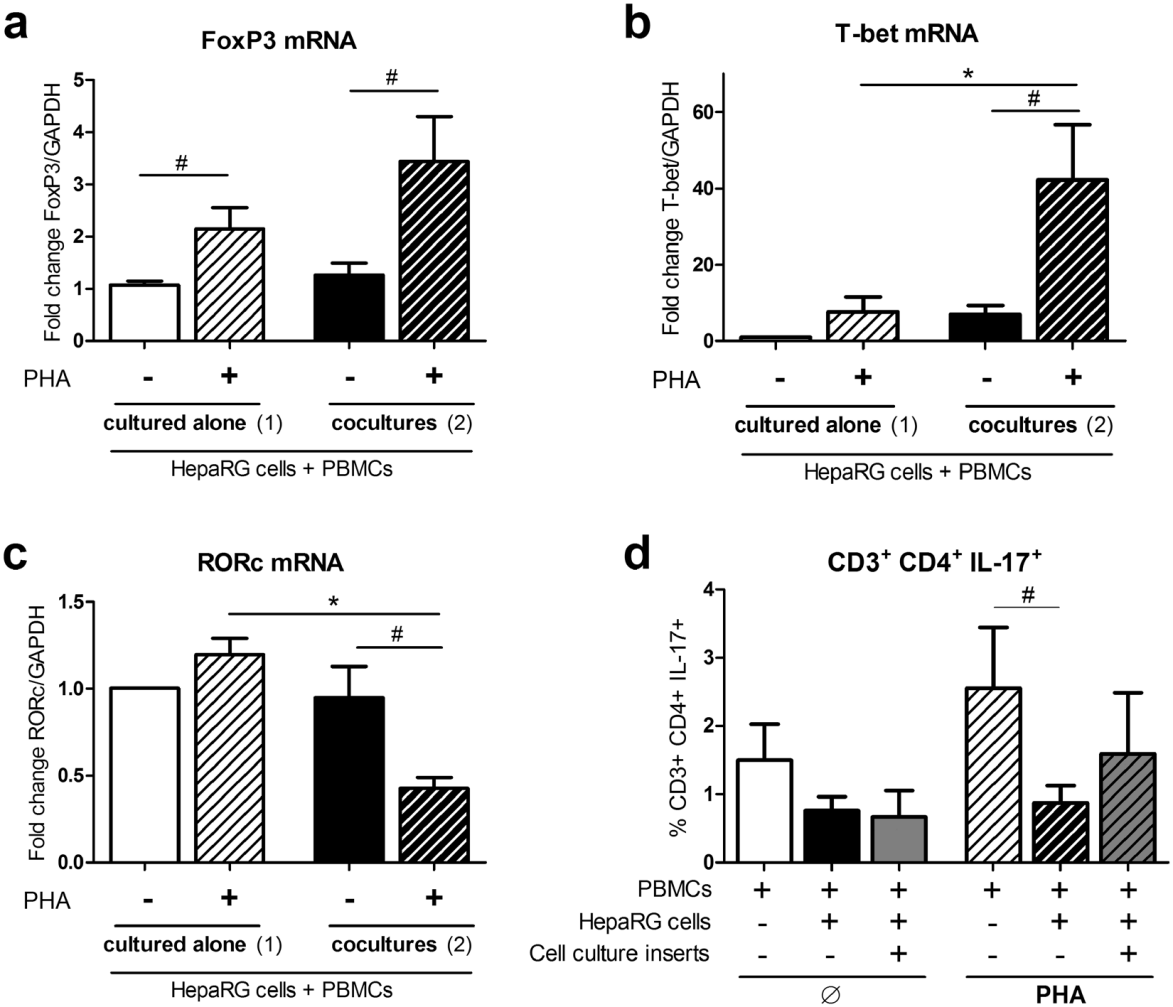
PBMC-HepaRG cell interactions up-regulate Tbet expression whereas RORc expression and IL-17^+^ CD4^+^ CD3^+^ cell frequency decrease in PHA-activated conditions. Human PBMCs and HepaRG cells were cultured alone or in co-cultures with or without cell culture inserts at a ratio of 5 PBMCs: 1 HepaRG cell in presence or not of PHA. (**a**–**c**) FoxP3, T-bet and RORc mRNA expression at 24 h was expressed as fold changes compared to non-activated conditions with PBMCs and HepaRG cells cultured alone. (**d**) Frequency of IL-17 positive CD4 T cells after 48 h of cultures. Cells were first gated on CD3 and CD4 expression. Data are the mean of 6 to 7 independent experiments ± SEM; Mann Whitney test, *p < 0.05 versus co-culture conditions; ^#^p < 0.05 versus non-activated conditions.

As IL-17, the signature cytokine of the Th17 cell, contributed to the induction of IL-6 and IL-8 in co-cultures, PBMC-HepaRG cell interactions may act on T cell polarization and secretion of specific T cell cytokines. Transcription factor mRNA expressions of Treg (FoxP3), Th1 (T-bet) and Th17 cells (RORc) were quantified in PBMCs and HepaRG cells cultured alone or together. PHA activation increased FoxP3 mRNA levels in both isolated cultures and co-cultures (p < 0.05) (Fig. 4a). In contrast, T-bet mRNA expression was significantly up-regulated whereas that of RORc was down-regulated in PHA-activated co-cultures compared to the other conditions (p < 0.05) (Fig. 4b,c). The frequency of CD3^+^ CD4^+^ IL-17^+^ cells was lower in PBMC-HepaRG cell co-cultures compared to PBMCs alone in presence of PHA confirming the effect on Th17 cells (Fig. 4d). Hepatocytes therefore contributed to T cell polarization in PHA-activated conditions by increasing Tbet expression and by reducing CD3^+^ CD4^+^ IL-17^+^ cell frequency.

### PBMC-HepaRG cell interactions decrease TNFα but not IL-10, IL-1β, IL-17 and IFNγ secretion

**Figure 5:**
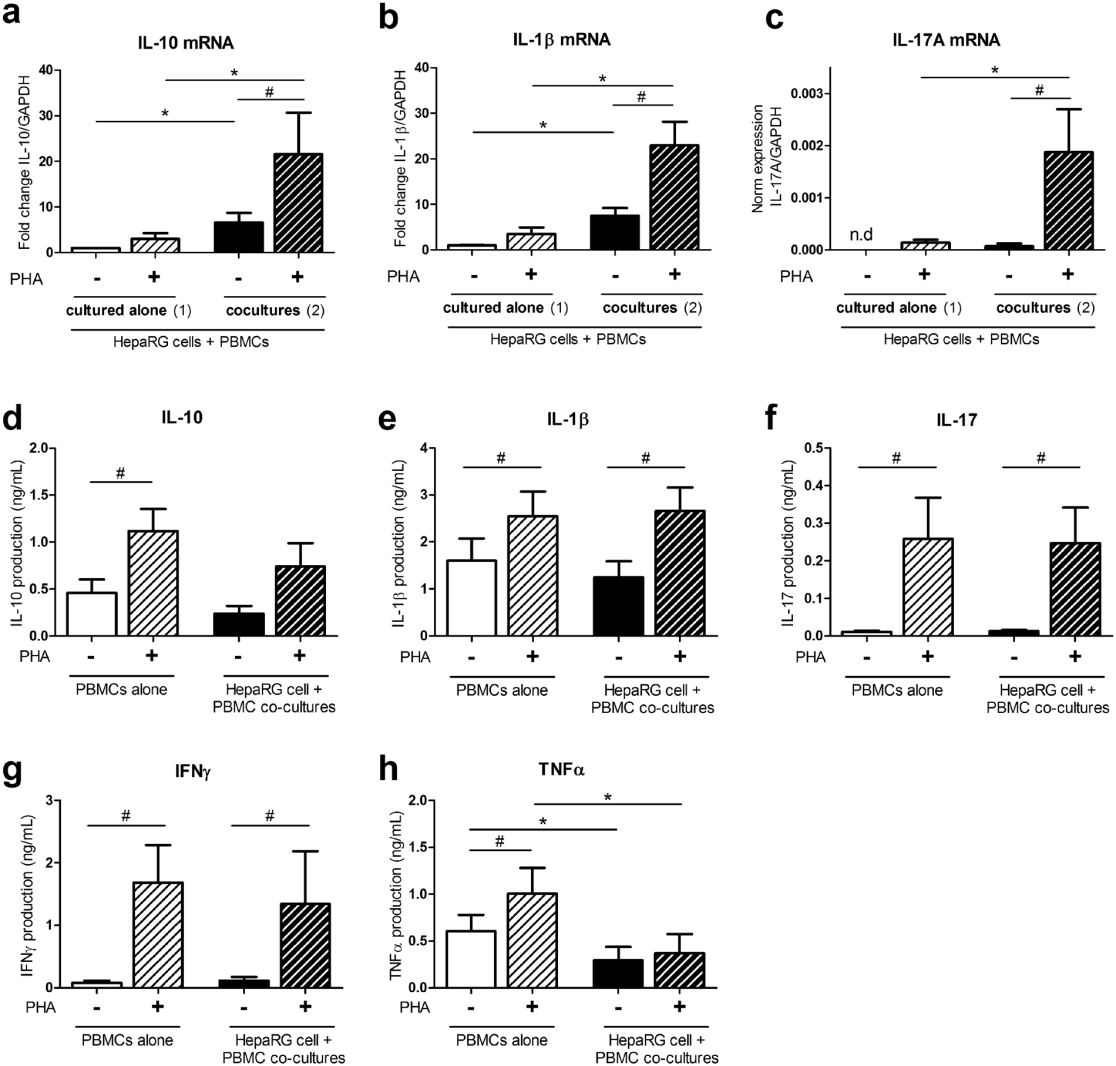
PBMC-HepaRG cell interactions decrease secretion of TNFα and had no effect on the IL-10, IL-1β, IL-17 and IFNγ release in PHA-activated condition. Human PBMCs and HepaRG cells were cultured alone or in co-cultures at a ratio of 5 PBMCs: 1 HepaRG cell in presence or not of PHA. (**a**,**b**) IL-10 and IL-1β mRNA expression at 24 h were expressed as fold changes compared to non-activated condition with PBMCs and HepaRG cells cultured separately. (**c**) IL-17A expression at 24 h was normalized to that of GAPDH. (**d**–**h**) IL-10, IL-1β, IL-17, IFNγ and TNFα supernatant levels were quantified by ELISA after 48 h of culture. Data are the mean of 6 to 11 independent experiments ± SEM; Mann Whitney test, *p < 0.05 versus co-culture conditions; ^#^p < 0.05 and ^##^p < 0.01 versus non-stimulated conditions.

Because hepatocytes can act on T cell polarization, mRNA and/or supernatant levels of the pro-inflammatory cytokines IL-1β, IL-17, IFNγ and TNFα and of the anti-inflammatory cytokine IL-10 were quantified in PBMC cultures in presence or not of HepaRG cells. mRNA levels of IL-10 and IL-1β increased with PBMC-HepaRG interactions with a higher induction in PHA-stimulated versus non-activated conditions (p < 0.05) (Fig. 5a,b). IL-17 mRNA level was strongly up-regulated in PHA-activated co-cultures compared to other conditions (p < 0.05) (Fig. 5c). Cytokine supernatant levels were then quantified in PBMC monocultures and PBMC-HepaRG cell co-cultures. In HepaRG cell cultures, cytokine production was too low compared to other culture conditions or not detectable (data not shown). Supernatant levels of IL-10, IL-1β, IL-17 and IFNγ were higher in PHA-conditions (p < 0.05) but similar between PBMCs alone and in co-cultures (Fig. 5d–g). In contrast, TNFα secretion was enhanced by PHA activation only when PBMC were cultured alone (p < 0.05) (Fig. 5h). Therefore, PBMC-HepaRG cell interactions induced gene expression of IL-10, IL-1β and IL-17 but not their actual secretion in the culture supernatants in PHA-activated conditions.

### Hepatocytes inhibit HLA-DR expression and proliferation of PHA-activated PBMCs

**Figure 6:**
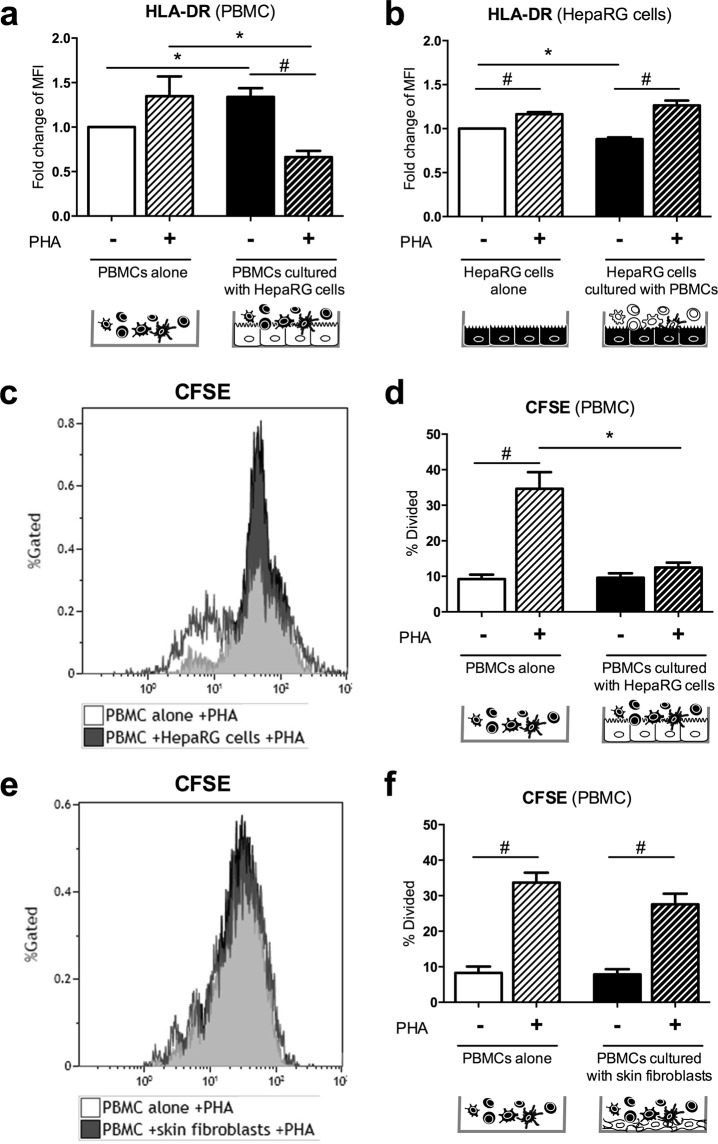
PBMC-HepaRG cell interactions reduce HLA-DR expression on PBMCs and the PHA-induced PBMC proliferation. Human PBMCs and HepaRG cells or skin fibroblasts were cultured alone or in co-cultures at a ratio of 5 PBMCs: 1 HepaRG cell or 1 skin fibroblast in presence or not of PHA. (**a**,**b**) HLA-DR expression was measured in HepaRG cells and PBMCs after 48 h of cultures by flow cytometry. (**c**–**e**) PBMCs were first labelled with CFSE and then cultured with/without HepaRG cells or skin fibroblasts and in presence or not of PHA for 3 days. Data are the mean of 6 to 7 independent experiments ± SEM; Mann Whitney test, *p < 0.05, vs. co-culture conditions; ^#^p < 0.05 vs. unstimulated conditions.

The effect of PBMC-HepaRG cell interactions on antigen presenting cell capacity to CD4^+^ T cells was next determined by looking at HLA-DR expression on both PBMCs and HepaRG cells as hepatocytes express major histocompatibility complex (MHC) class II in inflammatory conditions^13,14^. To distinguish PBMC and HepaRG cell population by cytometry, cells were stained with anti-CD45 antibody (Supplementary Fig. S1a). In PBMC, PBMC-HepaRG cell interactions enhanced HLA-DR expression by 1.4-fold (p < 0.05) versus PBMCs alone in non-activated conditions. In contrast, HLA-DR expression of PBMCs was down-regulated in PHA-activated co-cultures compared to other conditions (p < 0.05) (Fig. 6a). In HepaRG cells, PHA activation increased HLA-DR expression in both mono- and co-cultures (p < 0.05) whereas PBMC-HepaRG cell interactions rather decreased HLA-DR expression compared to HepaRG cells cultured alone in non-activated conditions (p < 0.05) (Fig. 6b).

The effect of PBMC-HepaRG cell interactions on PBMC proliferation was then investigated using carboxyfluorescein diacetate succinimidyl ester (CFSE) staining. As expected PHA activation enhanced the percentage of dividing cells (p < 0.05). However, this effect was suppressed in presence of HepaRG cells in the PBMCs cultures (Fig. 6c,d). To determine whether this inhibitory effect was specific of the hepatocytes, skin fibroblasts were co-cultured with PBMCS. PBMC-skin fibroblast interactions had no effect on the PHA-induced PBMC proliferation (Fig. 6e,f). To verify if the HepaRG cell inhibitory effect on PBMC division was related to cell death, cell viability was also determined. PBMC viability was reduced by 14% in PHA-activated PBMC monocultures (p < 0.01) but not in co-cultures (Supplementary Fig. S1c). HepaRG cell viability was also modulated by the presence of PBMCs activated or not with PHA. Viability of HepaRG cells especially decreased in PHA-activated co-cultures (Supplementary Fig. S1b). Therefore, HepaRG cells can reduce the antigen presenting cell capacity of PBMCs in PHA conditions as well as PBMC proliferation induced by PHA without affecting viability. After the early release of pro-inflammatory cytokines and chemokines induced by the PBMC-HepaRG cell interactions, HepaRG cells mediate anti-inflammatory effects on PBMCs, resulting in these two phases with rather opposite effects.

## Discussion

In this study, PBMC-HepaRG cell interactions enhanced immune cell recruitment and inflammation in liver by inducing IL-6 as well as IL-8, CCL20 and MCP-1 chemokine mRNA expression and/or secretion. In addition, hepatocytes modulated PBMC immune response by acting on T cell polarization and by reducing PBMC antigen presenting cell capacity and proliferation in activated conditions. PBMC-HepaRG cell interactions appear therefore to promote first an inflammatory response followed by a phase of inhibition of the PBMC immune response by hepatocytes.

To mimic the infiltration of immune cells during liver injury and inflammation, co-cultures between non-activated and activated human PBMCs and HepaRG cells were performed. Because healthy human liver biopsies are difficult to obtain, the HepaRG cells instead of primary human hepatocytes were used. The human HepaRG cells, which share many biological features with primary human hepatocytes^12,15^, represent a commonly used alternative tool. In our previous paper on the effect of cytokines on hepatocytes, the overall conclusion was similar between PHH and HepaRG cells. No significant contribution of alloreactivity has been detected previously in the same type of co-culture model^16^. In addition, in human skin fibroblast and PBMC co-cultures that are much more suspect of alloreactivity contribution regarding the risk of graft rejection, no difference between autologous and heterologous system has been observed in similar short-term cultutes^8^. The systemic inflammatory cytokine IL-6 is the major regulator of the release of acute-phase proteins, mainly produced by liver^9,17^. The first result showed that these interactions increased IL-6 secretion mainly through direct cell-cell contact in activated conditions.

Both acute and chronic inflammatory responses are characterized by the attraction of different immune cells induced by the local chemokine release. IL-8 is important for the mobilization and activation of neutrophils^18^ that are mainly involved in the early/acute response. CCL20 and MCP-1 chemokines attract various immune cells, which rather contribute to a more chronic phase. Here, PBMC-HepaRG cell interactions enhanced IL-8, CCL20 and MCP-1 expression and/or production in both non- and PHA-activated co-cultures. Up-regulation of IL-8 and MCP-1 expression has been also observed in the human monocyte cell line THP-1 and the human hepatoma HepG2 co-cultures compared to compared to THP-1 cell alone^19^. Through their effects on chemokine levels, these interactions will increase immune cell recruitment and therefore amplify cell interactions.

IL-17 and TNFα are two pro-inflammatory cytokines which has been implicated in several liver diseases^20–23^. As IL-17 and TNFα were found to induce in synergy IL-6 and IL-8 release by HepaRG cells, independently of IL-6^12^, their potential contributions to the induction of IL-6 and IL-8 secretion in activated PBMC–HepaRG cell co-cultures were assessed. To better mimic the *in vivo* conditions during chronic liver injury, PBMCs were pre-activated for one day and then co-cultured with HepaRG cells. Neutralization of IL-17 and/or TNFα inhibited IL-6 and IL-8 production in co-cultures without an additional inhibitory effect of the blockade of both IL-17 and TNFα. As previously demonstrated^12^, inhibition of IL-6 pathway did not reduce IL-8 secretion, confirming that this is an IL-6 independent effect. Soluble factor exchanges between PBMCs and HepaRG cells such as IL-17 and TNFα are thus important in the induction of IL-8, but to a lower extent of IL-6.

Hepatocytes-PBMC interactions may modulate PBMC phenotype and cytokine release^19^. In this study, Tbet expression was increased in PHA-activated co-cultures compared to PBMC alone whereas secretion of IFNγ, the signature cytokine of Th1 cells, was similar between these two conditions. Surprisingly, PBMC-HepaRG cell interactions decreased RORc expression and CD3^+^ CD4^+^ IL-17^+^ cell frequency whereas IL-17 mRNA expression increased in PHA-activated conditions. There were fewer Th17 cells in PHA-activated co-cultures, but they expressed higher quantity of IL-17 mRNA. Furthermore, IL-17 secretion remained similar between PBMC monocultures and co-cultures activated with PHA whereas TNFα release was lower in co-cultures. In addition, Treg transcription factor FoxP3 expression and IL-10 production were enhanced in PHA-activated cultures without difference between PBMCs cultured alone or with HepaRG cells. In contrast, another study showed that interactions between CD4^+^ T cells and mouse hepatocytes increased the frequency CD4^+^ FoxP3^+^ Treg cells and IL-10 secretion^24,25^. This induction of IL-10 and FoxP3^+^ Treg cells was even more pronounced when CD4^+^ T cells where co-cultured with hepatocytes from regenerated mouse liver pretreated with the lectin concanavalin A^24,25^. In our human co-culture system, gene expressions of IL-10, IL-1β and IL-17 were up-regulated in PHA-activated co-cultures but not actual IL-10, IL-1β and IL-17 secretion. Hepatocytes can thus act both at transcriptional and post-transcriptional levels by enhancing the transcription of several cytokines without increasing their release, possible under the influence of anti-inflammatory cytokines such as TGFβ.

Hepatocytes express MHC I and II and co-stimulatory molecules in inflammatory conditions^13,14^. HLA-DR expression of HepaRG cells decreased slightly in presence of non-activated PBMCs. In contrast, PHA-activation increased HLA-DR expression of HepaRG cells cultured alone or with PBMCs. Antigen-presenting cell ability of PBMCs to CD4^+^ T cells was also assessed. HLA-DR expression of PBMCs was up-regulated in non-activated co-cultures and down-regulated in PHA-activated co-cultures compared to monocultures. In activated conditions, HepaRG cells can inhibit initiation of antigen-specific immune response by reducing MHC II molecules of PBMCs. However, at the same time, MHC II expression on HepaRG cells was induced by PHA activation. This induction of antigen presentation by hepatocytes may influence the inflammatory response but was not sufficient to produce an effective immune response^14,26^.

As PBMCs proliferate upon activation, the effect of hepatocytes on PBMC proliferation was thus investigated. Previous studies have shown that murine and human hepatocytes are able to modulate T cell proliferation^16,24^. Here, in non-stimulated conditions, PBMC-HepaRG cell interactions had no effect on PBMC proliferation and death after 2 and 3 days of culture. However, as expected, PHA-stimulation enhanced PBMC proliferation but the presence of HepaRG cells reduced PHA-induced PBMC proliferation without affecting PBMC death. In contrast, PBMC proliferation induced by PHA was not impacted by PBMC-skin fibroblast interactions. Hepatocytes and skin fibroblasts have thus different effects on PBMC response, as observed in tolerance of graft rejection of these organs^2,3^. These immunosuppressive effects of hepatocytes on PBMCs can therefore contribute to the maintenance of immune tolerance in liver.

Cell interactions also influence cell survival and death. Increased survival of peripheral lymphocytes has been observed in presence of hepatocytes through the release of soluble factors^16^. In this study, PBMC viability was lower in PHA-activated PBMC monocultures compared to other conditions. HepaRG cell viability was also impacted by PHA and/or PBMCs. Indeed, PHA rather reduced viability of HepaRG cells cultured alone whereas the presence of PBMCs increased the frequency of viable HepaRG cells in non-activated co-cultures. Nevertheless, PHA-activated PBMCs decreased viability of HepaRG cells. Therefore, long-term exposure to activated PBMCs could lead to a massive HepaRG cell death and, consequently, a loss of the tolerogenic HepaRG cell activities. This situation probably occurs in chronic liver diseases that are characterized by hepatocyte death, sustained inflammation and the development of fibrosis driving to liver cirrhosis^23^.

In conclusion, hepatocyte-PBMC interactions induce a first pro-inflammatory phase characterized by the release of pro-inflammatory cytokines and chemokines. This phase is followed by a second phase with down-regulation of PBMC immune response. A better understanding of the role of these cell interactions in the hepatic immune response could lead to the identification of new therapeutic targets, that act in a sequential fashion. *In vivo* models of different liver disorders will be next useful to determine the nature and the contribution of these cell interactions for each pathology. Such understanding may provide new therapeutic options.

## Materials and Methods

### Cell culture

The human HepaRG cells were grown in William’s E medium (Sigma, St Louis, MO, USA) supplemented with 10% fetal bovine serum (Life Technologies, Carlsbad, USA), 2 mM L-glutamine (Eurobio, Les Ulis, France), 5 μg/mL insulin (Sigma), 50 μM hydrocortisone hemisuccinate (Serb, Paris, France), 50 U/mL penicillin and 50 μg/mL streptomycin (Eurobio). HepaRG cells were used after 15 days post-plating. Human skin fibroblasts were obtained from biopsies of non-lesional skin as previously described^8^. Fibroblasts were maintained in Dulbecco’s modified Eagle’s medium (Eurobio) supplemented with 10% fetal bovine serum (Life Technologies), 2 mM L-glutamine (Eurobio), 100 U/mL penicillin and 100 μg/mL streptomycin (Eurobio).

### PBMC isolation and co-culture assays

Whole blood samples were obtained from the Etablissement français du Sang. PBMCs were isolated by Ficoll-Hypaque (Eurobio) density gradient centrifugation. Cells were maintained in RPMI 1640 medium (Eurobio) supplemented with 10% human AB serum (Etablissement Français du Sang, La Plaine Saint-Denis, France), 2 mM L-glutamine (Eurobio). PBMCs were activated or not with 5 µg/ml phytohemagglutinin (PHA) (Sigma-Aldrich) and added on HepaRG cells or skin fibroblasts at a ratio of 5 PBMCs for 1 HepaRG cell or 1 skin fibroblast. This ratio was based on data from the literature^8,27^. For cell culture insert assays, HepaRG cells were cultured at the bottom of a culture plate well and PBMCs were placed in Falcon^®^ cell-culture inserts (Corning, NY, USA) with a small-pored membrane (0.4 µm) preventing direct cell-cell contacts but allowing the circulation of soluble factors. For the IL-17 and/or TNFα neutralization assays, PBMCs activated or not with PHA for 24 h were exposed to anti-IL-17 (R&D) at 10 µg/mL and/or anti-TNFα infliximab (Merck, Kenilworth, USA) at 10 µg/mL during 2 h before being added to the HepaRG cells. A monoclonal antibody against the BetV1 allergen (Dendritics, Lyon, France) was used as a control antibody at the same concentration.

Each individual signed an informed consent form. The protocol was approved by the committee for the protection of persons participating in biomedical research (number AC-2010-11-64).

### Quantitative real time-PCR

Total RNA was purified using RNeasy® Plus Mini kit (Qiagen, Hilden, Germany). cDNA was synthetized using the iScript™ kit (Bio-Rad, Hercules, CA, USA). PCR amplification was performed using the CFX96^TM^ Real time system instrument (Bio-Rad) with the iTaq^TM^ universal SYBR^®^ green supermix (Bio-Rad) and the Qiagen QuantiTect® primers. The expression of the genes of interest was normalized to the expression of the housekeeping GAPDH gene.

### Enzyme-linked immunosorbent assays

Supernatant cytokine concentrations were quantified with human ELISA kits according to the instructions of the manufacturer. IL-6 and IL-8 ELISA kits from Diaclone (Besancon, France) and CCL20, IL-17, TNFα, IFNγ, IL-1β and IL-10 ELISA kits from R&D system (Minneapolis, USA) were used.

### Flow cytometry

Cell phenotyping was performed on Navios flow cytometer (Beckman Coulter, Indianapolis, IN, USA). For CD3^+^ CD4^+^ IL-17^+^ cell staining, eFluor 450 labeled anti-CD3 antibody (clone UCHT1, 48-0038, eBiosciences, San Diego, CA, USA) and phytoerythrin (PE)-Cyanine7 labeled anti-CD4 antibody (clone RPA-T4, 25-0049, eBiosciences) were used for surface staining. After cell fixation with 2% paraformaldehyde (Sigma) for 15 min and cell permeabilization with 0.5% saponin (Sigma) for 20 min, the allophycocyanin (APC) labeled anti-IL-17A (clone eBio64DEC17, 17–7179, eBiosciences) were used for intracellular staining. For the other analysis, the PBMC population and the HepaRG cell population were distinguished by the use of the pacific blue (PB) labeled anti-CD45 (clone HI30, 304029, Biolegend, San Diego, CA, USA). PE labeled anti-HLA-DR (clone immu357, IM1639, Beckman Coulter) was used to stain HLA Class II. Corresponding isotypic antibodies labeled with the same fluorophores and from the same suppliers were used as controls. To track the PBMC proliferation, PBMCs were stained with CFSE as previously described by Quah B *et al*.^28^ Briefly, PBMCs (10.10^6^ cells/mL) were labelled with 1 μM CFSE for 5 min at room temperature, PBMCs were then washed and cultured for 3 days in presence or not of PHA. To investigate cell viability the fluorescein isothiocyanate (FITC) annexin V apoptosis detection kit (556547, BD, Franklin Lakes, NJ, USA) were performed according to the manufacturer’s instructions. Data were analyzed using Kaluza software (version 1.2, Beckman Coulter).

### Statistical analysis

Calculations were performed with GraphPad Prism version 5.01 software. Data are the mean of at least 3 independent experiments ± SEM. Statistical differences were analyzed using the Mann Whitney test. P values less than to 0.05 were considered significant.

## Supplementary information

PBMC hepatocyte interactions
