# Supplementary material for: Two phase kinetics of the inflammatory response from hepatocyte-peripheral blood mononuclear cell interactions
Source: Sci Rep. 2019 Jun 10;9:8378. doi: 10.1038/s41598-019-44840-w (PMC6557861; doi:10.1038/s41598-019-44840-w)
Supplement: Supplementary file 1 — PBMC hepatocyte interactions [file 41598_2019_44840_MOESM1_ESM.docx]

**Two phase kinetics of the inflammatory response**

**from hepatocyte-peripheral blood mononuclear cell interactions**

Audrey Beringer^1^, Jennifer Molle^2^, Birke Bartosch^2^, Pierre Miossec^1^





**Supplementary Figure S1: Effect of PBMC-HepaRG cell interactions on cell viability** PBMCs and HepaRG cells were cultured alone or in co-cultures at a ratio of 5 PBMCs : 1 HepaRG cell in presence or not of phytohemagglutinin (PHA). (a) Cells were labelled with pacific blue anti-CD45 antibody to distinguish by flow cytometry the PBMC population (CD45^+^) and the HepaRG cell population (CD45^-^). (b, c) Cells were stained with Annexin V (AnV)-FITC and propidium iodide (PI) to quantify the percentage of viable (AnV^-^ PI^-^), early apoptosis (AnV^+^ PI^-^) and dead (AnV^+^ PI^+^) cells by flow cytometry. Data are the mean of 8 independent experiments ± SEM; Mann Whitney test, *p<0.05 and **p<0.01, vs. monoculture conditions without PHA; #p<0.05, ##p<0.01 vs. monoculture conditions with PHA; §§p<0.01 vs. co-culture conditions without PHA.
